# *Akebia trifoliate (Thunb.) Koidz* Seed Extract Inhibits the Proliferation of Human Hepatocellular Carcinoma Cell Lines via Inducing Endoplasmic Reticulum Stress

**DOI:** 10.1155/2014/192749

**Published:** 2014-10-20

**Authors:** Wen-Li Lu, Hong-Yan Ren, Cao Liang, Yuan-Yuan Zhang, Ji Xu, Zhi-Qiang Pan, Xiao-Mei Liu, Zhong-Hua Wu, Zhao-Qin Fang

**Affiliations:** ^1^College of Basic Medical Science, Shanghai University of Traditional Chinese Medicine, No. 1200, Cailun Road, Shanghai 201203, China; ^2^Scientific Information Centre, Shanghai University of Traditional Chinese Medicine, No. 1200, Cailun Road, Shanghai 201203, China; ^3^Scientific & Technology Experimental Centre, Shanghai University of Traditional Chinese Medicine, No. 1200, Cailun Road, Shanghai 201203, China

## Abstract

Akebia Fructus has long been used for hepatocellular carcinoma (HCC) in China, while the molecular mechanism remains obscure. Our recent work found that *Akebia trifoliate (Thunb.) Koidz *seed extract (ATSE) suppressed proliferation and induced endoplasmic reticulum (ER) stress in SMMC-7721. The present study aimed to throw more light on the mechanism. ER stress occurred after ATSE treatment in HepG2, HuH7, and SMMC-7721 cells, manifested as ER expansion, and SMMC-7721 was the most sensitive kind in terms of morphology. Cell viability assay showed that ATSE significantly inhibited cells proliferation. Flow cytometry analysis indicated that ATSE leads to an upward tendency of G0/G1 phase and a reduced trend of the continuous peak after G2/M phase in HepG2; ATSE promoted apoptosis in HuH7 and a notable reduction in G0/G1 phase; ATSE does not quite influence cell cycles of SMMC-7721. Western blot analysis showed an increased trend of the chosen ER stress-related proteins after different treatments but nonsignificantly; only HYOU1 and GRP78 were decreased notably by ATSE in HuH7. Affymetrix array indicated that lots of ER stress-related genes' expressions were significantly altered, and downward is the main trend. These results suggest that ATSE have anticancer potency in HCC cells via partly inducing ER stress.

## 1. Introduction

According to the Word Cancer Report from IARC (International Agency for Research on Cancer), human hepatocellular carcinoma (HCC) is the sixth most common malignant cancer throughout the world and the third in China; the cases in China are more than half of the total worldwide [1, 2]. Surgical resection during early stages is still the dominant therapy for HCC. Unfortunately, most patients have already missed this opportunity by the time they see a doctor, and even patients who have been operated upon are at risk of recurrence, metastasis, and poor prognosis. On the other hand, most of the anticancer drugs may show severe side effects. Under these circumstances, the need of exploring new drugs in natural products, such as extracts of traditional Chinese herbs, becomes one promising alternative [3, 4]. In fact, in China, nearly 80% percent of patients suffering from HCC have taken herbs or herbal compounds to alleviate symptoms, relieve the side effects of radiotherapy or chemotherapy, improve the quality of life, and even extend life span [5–7].

Akebia Fructus is the near-mature dried fruit of plant* Akebia* and belongs to Lardizabalaceae family. The family is widely distributed over East Asia including China, Japan, and Korea. Akebia Fructus has long been used for HCC in China because of its traditional function of removing blood stasis and purging hard lump; it is also called “Bayuezha” due to ripe and crack in August [8]. There are three medicinal species, including* Akebia quinata (Thunb.) Decne*,* Akebia trifoliate (Thunb.) Koidz*, and* Akebia trifoliate (Thunb.) Koidz*.* var. australis (Diels.) Rehd*, whichare listed in the Chinese Pharmacopoeia [9]. In some experimental studies in vivo and vitro, the water extract of the whole fruit of* Akebia trifoliate*, the ethanol extract of* Akebia quinata* seed, or the separated ingredients showed antitumor activity [10–13], while the molecular mechanism remains obscure.

Akebia Fructus is always used as a whole fruit, which is composed of the pulp and the seed. In our recent research, extracts of different parts of* Akebia trifoliate (Thunb.) Koidz*, including the whole fruit, the pulp, and the seed, were used to treat SMMC-7721 HCC cells, respectively, and we unexpectedly found that seed part showed the strongest effect in inhibiting malignant proliferation and inducing endoplasmic reticulum (ER) stress [14–18], whilst no reference data on ER stress currently exist. In view of this, this further study aims to prepare an* Akebia trifoliate (Thunb.) Koidz* seed extract (ATSE) by n-butanol and throw more light on the molecular mechanism in a panel of three HCC cell lines, HepG2, HuH7, and SMMC-7721.

## 2. Material and Methods

### 2.1. Chemicals and Antibodies

Ethanol, Petroleum ether, ethyl acetate, and n-butanol are all AR grade and purchased from Sinophar Chemical Reagent Co., Ltd. (Shanghai, China). Trizol, G418, and 3-(4,5-dimethylthiazol-2-yl)-2,5-diphenyltetrazolium bromide (MTT) were purchased from Life Technologies (Carlsbad, CA, USA). Rabbit anti-SEC63 (1 : 800) polyclonal and rabbit anti-DNAJB11 (1 : 500) polyclonal were purchased from Sigma-Aldrich (St. Louis, MO, USA). Rabbit anti-HSP90AA1 (1 : 1000) was purchased from Stressgen (Enzo Life Sciences, NY, USA). Rabbit anti-monoclonal HYOU1 (1 : 5000) was purchased from Epitomics (Abcam, CA, USA). Mouse monoclonal anti-GRP78 (1 : 1000), mouse monoclonal anti-HSPA9 (1 : 1000), and mouse monoclonal anti-GAPDH (1 : 2500) were purchased from Santa Cruz Biotechnology (Santa Cruz, CA, USA). The secondary antibodies HRP Goat anti-mouse IgG and HRP Donkey anti-rabbit IgG were purchased from Biolegend (San Diego, CA, USA).

### 2.2. Plant Material

The whole fruit of* Akebia trifoliate (Thunb.) Koidz* was purchased from Kangqiao Herbal Pieces Co. Ltd. (Shanghai, China) in May 2013, which was produced in Zhejiang Province and authenticated by Lihong Wu, Institute of Chinese Meteria Medica, Shanghai University of Traditional Chinese Medicine; a voucher specimen (number 130319) was deposited in the standardization lab of this institute.

### 2.3. Preparation of* Akebia trifoliate (Thunb.) Koidz* Seed Extract (ATSE)

The seed was peeled off from the whole fruit and dried at 60°C for 24 hours; dried seed (800 g) was smashed and soaked in 75% ethanol for 2 hours at room temperature and then extracted by 8 L 75% ethanol reflux at 80°C for 2 hours and filtered by gauze; the filtration residue was extracted again under the same condition; all resulting filtrations were combined and concentrated by Rotavapor (BÜCHI Labortechnik, Switzerland) under reduced pressure; 2700 mL concentrated extract was obtained, followed by successive extraction with the same volume of Petroleum ether, ethyl acetate, and water-saturated n-butanol, three times in each solvent; this procedure resulted in three extracts; the n-butanol soluble extract was further concentrated by Rotary Evaporator (IKA, Germany) at 60°C (20–40 rpm); 310 mL extractum was obtained and then freeze-dried to 33.26 g power, which is simply called ATSE in experiment. The extract yield was 4.26% (w/w). ATSE was diluted to 0.5 g/mL by distilled water and then dissolved in the RPMI 1640 culture medium to 10 mg/mL and finally filtered through a 0.45 *μ*m filter for use.

### 2.4. Cell Lines and Culture

HepG2, HuH7, and SMMC-7721 cell lines were obtained from Chinese Academy of Sciences (Shanghai, China), cells were maintained in RPMI 1640 medium supplemented with 10% fetal bovine serum (FBS), 100 U/mL penicillin, and 100 *μ*g/mL streptomycin. RPMI 1640 medium, Trypsin, penicillin, and streptomycin were all purchased from Hyclone (Thermo scientific, UT, USA). These cells were routinely cultured in humidified 95% air and 5% CO_2_ at 37°C.

### 2.5. Cell Morphological Assessment

Cells were split and seeded in 96-well and 6-well plates, 18–24 hours after seeding, and when the confluency was about 70%, G418 or ATSE was added to four different groups according to the requirement: untreated control; G418 group (0.45 mg/mL); ATSE group (0.625 mg/mL); the combination group was composed of half dose of each (0.225 mg/mL G418 plus 0.31 mg/mL ATSE). 72 hours after treatment, cells in 96-well plates were used for MTT assay, and the cell morphology of 6-well plates was firstly examined under an inverted phase contrast microscope (Olympus CKX41, Tokyo, Japan), then for the other different assays.

### 2.6. Cell Viability Assay

Cell survival was assessed using an MTT assay at 72 hours after treatment; the culture medium of 96-well plate was removed and replaced with 0.5 mg/mL MTT solution. After 4 h incubation at 37°C, this solution was removed and the resulting blue formazan was solubilized in 100 *μ*L DMSO; then the optical density was read at 490 nm using a microplate reader (BioTek Synerge 2, BioTek Instruments, VT, USA). Cell viability was expressed as a percentage of the control.

### 2.7. Flow Cytometry Analysis of Cell Cycle

Treatments of cells were performed according to the protocol of the PI detection kit (KeyGEN Biotech, Shanghai, China). Briefly, cells were harvested and resuspended in cold PBS at a concentration of 1 × 10^6^ cells/mL and then fixed by 3 times volume of cold anhydrous ethanol (final concentration is 75%) and kept at 4°C for 2 hours; ethanol was then removed and resuspended in 500 *μ*L buffer A, followed by addition of RNase A (final concentration was 0.25 mg/mL) and 5 *μ*L PI solution, and then incubated for 30 min at room temperature in the dark; finally analysis was performed by using a Flow Cytometer (BD FACS Calibur, BD Biosciences, CA, USA).

### 2.8. Western Blot Analysis

Cell lysates were harvested in RIPA buffer (50 mM Tris (pH 7.4), 150 mM NaCl, 1% NP-40, 0.5% sodium deoxycholate, and 0.1% SDS). The whole cell lysates were centrifuged at 12,000 rpm for 15 minutes at 4°C; then the supernatant was collected and protein concentration was measured by Pierce BCA Protein Assay Kit (Thermo scientific, UT, USA). Cell lysates (20 *μ*g) were resolving on 6–8% SDS-PAGE followed by transfer onto 0.45 *μ*m PVDF membrane (Merk millpore, MA, USA). Subsequently, membranes were incubated with 5% skimmed milk in TBST for 1 h, followed by probing with the primary antibody overnight at 4°C. After washing with TBST, the membranes were incubated for 1 h at room temperature with HRP-conjugated secondary antibodies; bands were visualized using BeyoECL Plus western blot detection system (Beyotime, Shanghai, China). Results of the Western blot assay reported here are representative of three experiments; glyceraldehyde-3-phosphate dehydrogenase (GAPDH) was used as internal control.

### 2.9. Affymetrix Array

Total RNA was isolated from cells with Trizol according to manufacturer's protocol. The qualities of all RNA samples were monitored: the absorbance at 260 nm and 280 nm was measured by NanoDrop 1000 (Thermo Fisher Scientific, MA, USA) and acceptable A260/280 ratios were in the range of 1.7–2.1; the RNA 6000 Nano kit (Agilent Technologies, CA, USA) was used to detect RIN (RNA integrity number) and 28S/18S ratios by Agilent 2100 Bioanalyzer (Agilent Technologies, CA, USA); the former was approximately 9.2 and the latter were approaching 2 : 1. High-quality total RNA was used as a starting material for making total RNA/Poly-A RNA controls and was mixed using a GeneChipR Poly-A RNA Control Kit (Affymetrix, CA, USA). Then cDNA was synthesized using an Ambion WT Expression Kit (Affymetrix, CA, USA), followed by ssDNA fragmentation and labeling of fragmented ssDNA with GeneChip WT Terminal Labeling Kit (Affymetrix, CA, USA). The biotin-labeled fragmentations were hybridized to a gene chip (Affymetrix HuGene 1.1 Sense Target Array) at 48°C for 16 h. Following hybridization, the chips were washed and stained in the GeneAtlas Fludic Station (Affymetrix, CA, USA). Then the arrays were put into the GeneAtlas Imaging Station (Affymetrix, CA, USA). Microarray data were analyzed by Affymetrix Expression Console (Affymetrix, CA, USA). The data were normalized using the iterative PLIER default protocol. Changes in gene expressions were analyzed and compared with untreated control and the criteria for positive fold change are greater than 1.5-fold increase or decrease.

### 2.10. Statistical Analysis

Data were analyzed using the IBM statistics SPSS 19.0 software. The results are presented as means ± standard deviation (S.D.) and the comparison between groups was analyzed by one-way analysis of variance (ANOVA). A value of *P* < 0.05 was considered as statistically significant. All experiments were performed for a minimum of three times.

## 3. Results

### 3.1. ATSE Cause Different Morphological Changes in HCC Cell Lines

**Figure 1:**
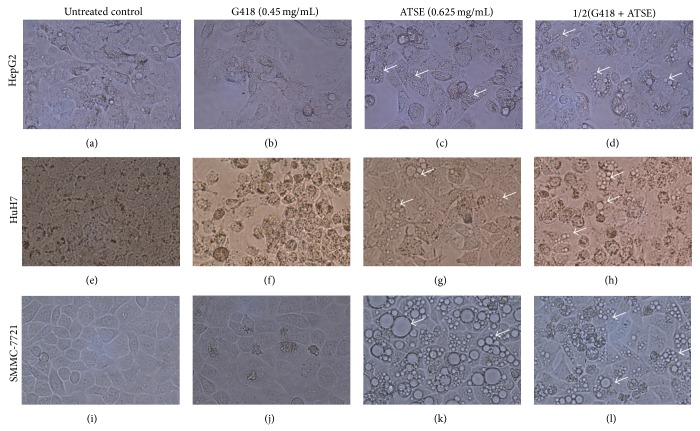
ATSE cause different morphological changes in HCC cell lines. G418 (0.45 mg/mL), ATSE:* Akebia trifoliate* seed extract (0.625 mg/mL), and 1/2(G418 + ATSE): a combination of half dosage of each accordingly. (a–d) Images of HepG2 cells after different treatments, (e–h) images of HuH7 cells after different treatments, and (i–l) images of SMMC-7721 cells after different treatments. White arrowhead means different degree of ER expansion within cells. These images were taken under the 400x inverted phase contrast microscope.

The morphological changes induced by ATSE were observed. HepG2 cells were not sensitive to 0.45 mg/mL G418 (Figure 1(b)), while mild endoplasmic reticulum stress (see the white arrowhead) was induced by 0.625 mg/mL ATSE (Figure 1(c)); the effect of the combination of half doses of each (Figure 1(d)) was similar to that in Figure 1(c). In contrast to HepG2 cells, 0.45 mg/mL G418 caused obvious morphological changes in HuH7 cells, including a decreased number of cells, increased blank area, and even emergence of the apoptotic body in a couple of cells (Figure 1(f)); 0.625 mg/mL ATSE caused mild ER stress (Figure 1(g)); the effect of the combination group (Figure 1(h)) was like the overlay of Figures 1(f) and 1(g). As to SMMC-7721 cells, 0.625 mg/mL ATSE resulted in remarkable ER stress, displayed as different degree of ER expansion (Figure 1(k)); in the combination group (Figure 1(l)), since the ATSE dose was cut by half, the ER stress was comparatively lighter.

In brief, ATSE induced ER stress in these three HCC cell lines but the degree varied and it seemed that SMMC-7721 cells were the most sensitive kind.

### 3.2. ATSE Suppress Cell Viability in HCC Cell Lines

**Figure 2:**
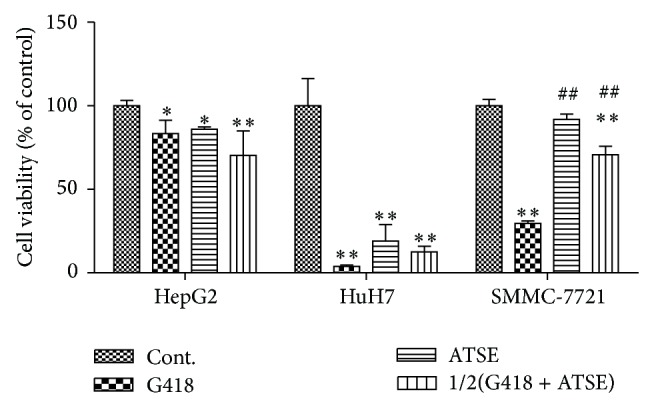
ATSE suppress cell viability in HCC cell lines. Cell viability was tested by MTT. G418 (0.45 mg/mL), ATSE:* Akebia trifoliate* seed extract (0.625 mg/mL), and 1/2(G418 + ATSE): a combination of half dosage of each accordingly. The results are expressed as mean ± S.D. (*n* = 4). ^**^ *P* < 0.01 and ^*^ *P* < 0.05 (versus control group); ^##^ *P* < 0.01 and ^#^ *P* < 0.05 (versus G418 group).

To assess the effect of ATSE on cell viability, MTT assay was performed. As shown in Figure 2, compared to the untreated control, cell viability decreased significantly but varied in degree: values of ATSE groups were reduced to 85.9% in HepG2, 19.1% in HuH7, and 91.8% in SMMC-7721 cells, respectively; besides, in G418 groups, values were reduced to 83.3% in HepG2, 3.8% in HuH7, and 29.6% in SMMC-7721 cells, respectively; in the combination groups, values were reduced to 70.3% in HepG2, 12.5% in HuH7, and 70.7% in SMMC-7721 cells, respectively. On the other hand, in HepG2 and HuH7 cells, ATSE and the combination groups exhibited no significant difference compared to G418, but in SMMC-7721 cells, the values of these two groups were notably higher than that of G418.

### 3.3. Different Cell Cycle Influences of ATSE on HCC Cell Lines

To figure out whether the proliferation inhibition is due to cell cycle arrest, the cell cycle analysis by Flow cytometry was performed, and these HCC cell lines showed different features as shown below.

**Figure 3:**
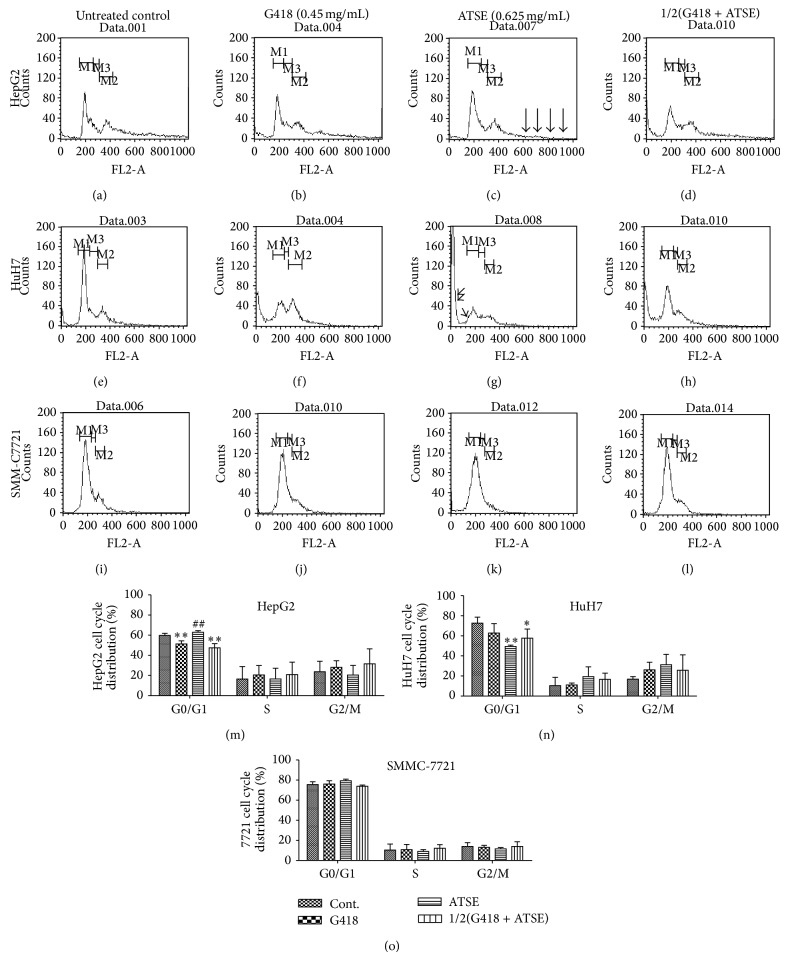
Different cell cycle influences of ATSE on HCC cell lines. Cell cycle analysis was performed by Flow Cytometry. G418 (0.45 mg/mL); ATSE:* Akebia trifoliate* seed extract (0.625 mg/mL); 1/2(G418 + ATSE): a combination of half dosage of each accordingly. (a–d) Images of HepG2 cells after different treatments, (e–h) images of HuH7 cells after different treatments, (i–l) images of SMMC-7721 cells after different treatments, (m) HepG2 cell cycle distribution, (n) HuH7 cell cycle distribution, and (o) SMMC-7721 cell cycle distribution. M1: G0/G1 phase, M2: G2/M phase, and M3: S phase. (↓↓↓↓) continuous peak after G2/M phase after ATSE treatment, (↓↓) apoptotic body peak, and (↓) apoptotic cell peak. The results are expressed as mean ± S.D. (*n* = 3). ^**^ *P* < 0.01 and ^*^ *P* < 0.05 (versus control group); ^##^ *P* < 0.01 and ^#^ *P* < 0.05 (versus G418 group).

Firstly, In HepG2 cells, at 72 hours after G418 incubation, the percentage of cells in G0/G1 phase decreased significantly to 52.9% versus 59.7% of the control (Figures 3(b) and 3(m)), while the percentage of same phase for ATSE was 62.9% and slightly higher than the control, albeit nonsignificantly (Figures 3(c) and 3(m)), which possibly indicated various action modes between ATSE and G418 on HepG2 cells; furthermore, it seemed that the amount of the continuous peak after G2/M phase was reduced (see black arrowheads in Figure 3(c)); this could be another aspect of ATSE (Figures 3(a) and 3(c)). An apoptotic peak appeared in the combination group and the percentage reduced to 47.5% notably (Figures 3(d) and 3(m)).

In HuH7 cells (Figures 3(e) to 3(h)), 72 hours after cultivation, apoptosis occurred among all groups before G0/G1 phase, especially in ATSE group. Apoptotic body peak (see double arrowheads, Figure 3(g)) and apoptotic cell peak (see single arrowhead, Figure 3(g)) can be seen, which is typically different from the other two kinds and corresponding to MTT assay data (Figure 2). This unique feature also influences its cell cycles, including the percentage in G0/G1 phase which decreased significantly after ATSE or combined treatment, specifically 49.4% for ATSE and 57.7% for the latter, and a corresponding increasing trend in S phase and G2/M phase (Figures 3(g), 3(h), and 3(n)). The percentage in G0/G1 phase of G418 reduced to 62.8% but there is no statistical difference versus control group (*P* > 0.05; Figures 3(f) and 3(n)).

Unlike HepG2 or HuH7, basically ATSE does not influence much cell cycles of SMMC-7721 (Figures 3(i) to 3(l)). There is no statistical difference between all groups (*P* > 0.05; Figure 3(o)).

### 3.4. Effect of ATSE on the Protein Expression of ER Stress-Related Biomarkers

**Figure 4:**
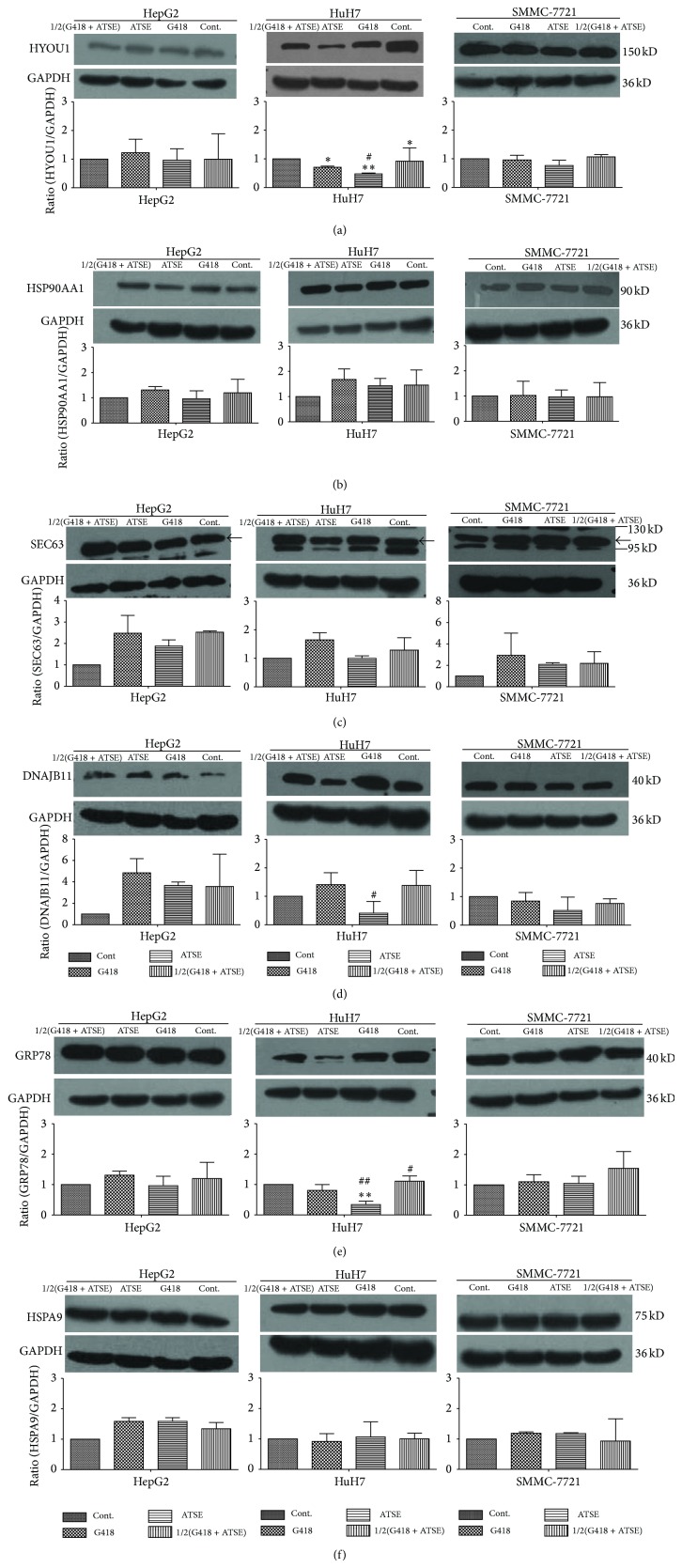
Effect of ATSE on the protein expression of ER stress-related biomarkers. The relative expression of ER-stress biomarkers at protein level after 72-hour treatment. GAPDH was used as internal control. G418 (0.45 mg/mL); ATSE:* Akebia trifoliate* seed extract (0.625 mg/mL); 1/2(G418 + ATSE): a combination of half dosage of each accordingly. (a) relative protein expression of HYOU1, (b) relative protein expression of HSP90AA1, (c) relative protein expression of SEC63, (d) relative expression of DNAJB11, (e) relative protein expression of GRP78, and (f) relative protein expression of HSPA9. The values given are the mean ± S.D. (*n* = 3); ^**^ *P* < 0.01 and ^*^ *P* < 0.05 (versus control group); ^##^ *P* < 0.01 and ^#^ *P* < 0.05 (versus G418 group).

To further clarify the molecular mechanism of ATSE induced ER stress in HepG2, HuH7, and SMMC-7721 cells, some ER stress-related proteins were chosen and examined by Western blot analysis. As indicated in Figure 4, compared with the untreated control, most of the proteins expressions displayed an upward tendency after different treatments, albeit nonsignificantly (*P* > 0.05). However, in HuH7 cells, ATSE decreased the protein expression of HYOU1 and GRP78 significantly (Figures 4(a) and 4(e)). On the other hand, compared with G418, most of the proteins expressions in response to ATSE indicated a reduced trend, and HYOU1, GRP78, and DNAJB11 were reduced notably in HuH7 (Figures 4(a), 4(d), and 4(e)).

### 3.5. Expression of ER Stress-Related Genes in HepG2 Cells

For full characterization of the molecular mechanism of ATSE, Affymetrix array was used. 82 ER stress-related genes were selected one by one manually from NCBI gene database. There were significant changes in the levels of gene expression in 8 of 82 genes (Table 1); HSP90AA1 was included and upregulated by ATSE significantly.

### 3.6. Expression of ER Stress-Related Genes in HuH7 Cells

There were significant changes in the levels of gene expression in 38 of 82 genes (Table 2); HSP90AA1 was included and upregulated by ATSE significantly. On the other hand, HSPA9, DNAJB11, SEC63, HYOU1, and GRP78 were all downregulated notably. Furthermore, most of the genes were downregulated.

### 3.7. Expression of ER Stress-Related Genes in SMMC-7721 Cells

There were significant changes in the levels of gene expression in 19 of 82 genes (Table 3). SEC63, DNAJB11, HSP90AA1, and GRP78 were all downregulated notably. It is similar to HuH7 that most of the genes were downregulated notably.

### 3.8. Expression of the KEGG Integrin-Mediated Cell Adhesion Pathway for HepG2 Cells

Affymetrix array data also presented genes expressions information of KEGG integrin-mediated cell adhesion pathway, which helps to explain the effect of ATSE on HepG2 cells. There were significant changes in 6 of 89 genes, 4 were upregulated and 2 were downregulated (Table 4).

### 3.9. Expression of the KEGG Apoptosis Pathway for HuH7 Cells

89 genes in the KEGG apoptosis pathway were displayed in Affymetrix array data. There were significant changes in the levels of gene expression in 6 of 89 genes, 5 were upregulated and 1 was downregulated (Table 5).

## 4. Discussion

Akebia Fructus has been long used for HCC in TCM in China, in the manner of one member in herbal formula under most circumstances. The data from clinical epidemiological investigation on 2060 HCC cases [19], and retrospective analysis on the published papers containing the use of herbal formulas for HCC, indicated that the frequency for Akebiae Fructus is up to nearly 43% [20], which means that Akebiae Fructus is popular in herbal formula and plays an important role and also means that it is worthwhile to reveal the underlying molecular mechanism of how this herb works and a possibility of a new anticancer agent.

Therefore, in our recently work [14–18], we chose* Akebia trifoliate (Thunb.) Koidz*, one of the three medicinal species in China, to carry out research. Considering Akebia Fructus is always used as a whole fruit, we separated the fruit into two parts, the pulp and the seed, to observe the effect on SMMC-7721 cells, and the result was beyond expectation. We found that* Akebia trifoliate* seed extract (ATSE) could suppress the proliferation and caused notable ER stress in SMMC-7721 cells, which suggested ATSE could inhibit proliferation and probably via inducing ER stress. To further investigate this phenomenon, HepG2, HuH7, and SMMC-7721 cells were used and the dose of ATSE was according to our previous work [14–18]. In view of G418 being a commonly used cytotoxic antibiotic that can suppress proliferation of many cancer cells and presumable advantages of combined therapy, G418 and combination groups were also set up. The combination group is composed of half doses of each.

Our data exhibited that ATSE leads to different degrees of ER stress in these HCC cell lines and a decrease in cell viability (Figures 1 and 2). In fact, a lot of previous work has been done to investigate the herbal medicine on HCC, and several mechanisms of action have been clarified [4], such as cytotoxic activities against cancer cell lines [21, 22], while the effect that related ER stress to HCC cells was relatively rarely mentioned. On the other hand, the endoplasmic reticulum has been posited as a potential anticancer target [23]. Indeed some anticancer chemotherapeutic agents have been shown to induce ER stress, such as the proteasome inhibitor bortezomib [24], as well as the cannabinoids [25]. However, most of these kinds of research did not provide visual morphological evidence of ER stress, while our data was presented intuitively and suggested that SMMC-7721 is the most sensitive in terms of morphology (Figure 1).

As shown in Figure 3, the cell cycle analysis by FCM was performed to find out the relevance of cell cycle and proliferation inhibition. Basically, not so many notable changes in statistical data were observed. On the other hand, in contrast to the uncertain cell cycle alteration, other information, just like cells which behaved differently in response to ATSE, cannot be ignored. Firstly, in HepG2 cells, a continuous peak can be seen after the G2/M phase (Figure 3(a)) and it seemed that the amount of peak was reduced by ATSE (Figure 3(c)), implying that ATSE may reduce the adhesion between HepG2 cells, which is meaningful for fewer tumor metastases. This speculation was based on the following: contact inhibition does not happen to HepG2, so cells could grow overlapped. It is difficult to separate cells to a single one in vitro, which is distinguished from the other two kinds. Therefore, when the PI stained HepG2 cells pass through FCM, the continuous peak after G2/M phase formed. Another reference is gene expression in the integrin-mediated cell adhesion pathway (Table 4). Further experiments such as the cell adhesion assay can be considered. Secondly, unlike the other two kinds, HuH7 cells proliferate very quickly. Apoptosis occurs spontaneously in the absence of ATSE (Figure 3(e)) and intensified in the presence of ATSE (Figure 3(g)). Thus, ER stress-mediated apoptosis is possibly involved; many genes expressions in the apoptosis pathway are upregulated by ATSE as well (Table 5). All these data reveal that the distinction exists in ATSE against different HCC cells. In addition, cell cycle regulatory proteins can be considered in further research.

Besides morphological changes and cell cycle alterations, ER stress is often accompanied with expression changes of a lot of genes and proteins, or called as biomarkers. Generally, this event started from three transmembrane sensors in UPR (unfolded protein response) pathway [26, 27], namely, ATF6, IRE1, and PERK, and a lot of downstream molecules, such as Grp78 and DDIT3. Some effective ingredients from herb, such as Tanshinone IIA, have shown the effect of induction of ER stress on prostate cancer cells, and increased expression of Grp78 was identified [28]. ER stress was activated by Tubeimoside-1, a triterpenoid saponin extracted from* Bolbostemma paniculatum*, with an increased expression of DDIT3 in human cervical carcinoma cells [29], while our previous data indicated that ATSE selectively suppressed mRNA expression of many ER stress-related genes in SMMC-7721 cells, especially of Hyou1, Hsp90aa1, Sec63, Dnajb11, Grp78, and Hspa9. To understand more about this, further proteins expressions were monitored by WB, genes' profiles were assessed by Affeymetrix array as well and more cell lines were used besides SMMC-7721. First, in HepG2 and SMMC-7721 cells, ATSE led to an increased trend of these chosen proteins versus control, but not significantly, while in HuH7 cells, Hyou1 and Grp78 were notably downregulated (Figure 4). Secondly, a lot of genes showed significant changes versus control, and downward is the main trend (Tables 1, 2, and 3). As to the six selected genes, only HSP90AA1 was upregulated In HepG2 and HuH7, and the others were all downregulated in all cell lines. Combining the expression feature of proteins and genes, one relatively reasonable explanation is that the alteration in the ER stress event depends on cell's status, as many factors could induce ER stress, and degree varies. When cells are under adaptation circumstances, UPR pathway works and a lot of molecules were mobilized and against stress successfully, such as Grp78 which helps to maintain ER integrity. However, if stress is beyond cells' tolerance or stress continues, apoptosis will be one possible outlet and several molecules such as DDIT3 will be designated to mediate this process [27, 30], and subsequently more molecules will take part in the event or quit, which make the situation complex.

Furthermore, even Affeymetrix array presented such a comprehensive gene expression profile; how ER stress was induced and how these genes or proteins are influenced by ATSE, active or passive, directly or indirectly remain unclear. Therefore, besides the continued analysis of these data, further investigations, such as cotreatment with some mechanism-known ER-stress-induced agents and such as genes knocked down by RNA interference, are beneficial to know more about how ATSE works and the role of those significantly changed genes or proteins.

The phytochemistry of ATSE is not discussed in the present work, for the main ingredients are almost clear, which are triterpenoid saponins. Since the work of Ryuichi et al. in the last century [31, 32], there are around 90 triterpenoid saponins which have been discovered in* Akebia Decne* so far [33, 34], including* Akebia trifoliate (Thunb.) Koidz* and other medicinal species; medicinal parts involve fruit, seed, stem root, and other sections. However, it remains important to further characterize new biological activities of known ingredients.

The effect of drug combination is also worthy of attention. As shown in the MTT result (Figure 2), the cell viability of the combination group decreased significantly compared to the control, which was consistent in all cell lines, and the level was lower than G418 and the ATSE group in HepG2 cells as well as the apoptotic body peak before G0/G1 phase responded to this (Figure 3(d)). Overall though, we speculate that if ATSE combined with a cytotoxic antibiotic, it would make sense to affect a dosage reduction of cytotoxic antibiotics or herb preparation, for therapeutic efficacy to improve, for side effects to be reduced, and to create more opportunities for patients suffering from cancer.

In summary, our data demonstrated that the* Akebia trifoliate (Thunb.) Koidz* seed extract (ATSE) has a certain effect on proliferation inhibition in HepG2, HuH7, and SMMC-7721 cell lines and ER stress induction involved. It provides a promising candidate therapeutic agent anti-HCC, and further studies are required.

## Figures and Tables

**Table 1:** List of ER stress-related genes with significant expression in HepG2 cells (fold change ≥1.5-fold).

Probe set ID	Gene symbol	Control	G418	ATSE	1/2(G418 + ATSE)	ATSE/control
8152628	DERL1	199	336	355	694	1.78
7954196	MGST1	733	958	1305	1663	1.78
7995895	HERPUD1	714	1239	1166	2351	1.63
**7981335**	**HSP90AA1**	**1263**	**2402**	**1946**	**3732**	**1.54**
7956593	OS9	315	357	475	407	1.51
8156838	SEC61B	528	426	348	631	−1.52
8080084	MANF	495	587	320	791	−1.55
8075182	XBP1	758	583	352	343	−2.16

G418 (0.45 mg/mL); ATSE: *Akebia trifoliate* seed extract (0.625 mg/mL); 1/2(G418 + ATSE): a combination of half dose of each accordingly.

**Table 2:** List of ER stress-related genes with significant expression in HuH7 cells (fold change ≥1.5-fold).

Probe set ID	Gene symbol	Control	G418	ATSE	1/2(G418 + ATSE)	ATSE/control
7954196	MGST1	479	815	1026	720	2.14
8024754	CREB3L3	171	55	353	69	2.06
7967563	UBC	2502	4281	4550	7132	1.82
**7981335**	**HSP90AA1**	**1153**	**3916**	**1725**	**3257**	**1.50**
**8114455**	**HSPA9**	**1116**	**1499**	**749**	**1115**	−**1.50**
7949383	SYVN1	326	235	208	224	−1.57
7964460	DDIT3	239	137	151	332	−1.58
8125295	ATF6B	202	49	117	82	−1.73
8080084	MANF	268	490	152	351	−1.77
8075182	XBP1	820	705	448	596	−1.83
8091458	SERP1	561	1050	303	635	−1.85
8137526	INSIG1	214	356	114	290	−1.88
7916432	DHCR24	2595	560	1278	280	−2.03
8138108	KDELR2	1272	1329	622	605	−2.04
8160914	VCP	423	764	200	227	−2.11
7989619	PPIB	1939	2258	917	2377	−2.11
8066889	STAU1	398	248	187	98	−2.12
8128111	UBE2J1	474	344	199	183	−2.38
7916120	TXNDC12	817	404	330	172	−2.48
7958644	ATP2A2	492	570	186	206	−2.65
8026106	CALR	1329	1694	497	1060	−2.67
8051998	MCFD2	411	507	149	213	−2.76
7995895	HERPUD1	915	870	297	960	−3.08
**8084634**	**DNAJB11**	**279**	**947**	**89**	**529**	−**3.14**
7906819	ATF6	268	361	80	174	−3.33
8041967	ERLEC1	275	363	77	109	−3.57
**8128650**	**SEC63**	**982**	**1218**	**265**	**504**	−**3.71**
8135480	DNAJB9	256	431	53	351	−4.86
**7952145**	**HYOU1**	**492**	**757**	**97**	**266**	−**5.06**
7956593	OS9	1008	857	195	383	−5.17
8046759	DNAJC10	452	202	77	27	−5.87
8023561	LMAN1	941	1298	159	448	−5.93
7980547	SEL1L	333	295	46	68	−7.27
**8164165**	**GRP78**	**1834**	**2581**	**241**	**1442**	−**7.62**
7904881	PDIA3P	1192	1261	131	644	−9.11
7958130	HSP90B1	1813	2793	198	1396	−9.17
7983274	PDIA3	1181	1226	119	602	−9.95
8095628	ALB	3131	1860	63	435	−49.59

G418 (0.45 mg/mL); ATSE: *Akebia trifoliate* seed extract (0.625 mg/mL); 1/2(G418 + ATSE): a combination of half dose of each accordingly.

**Table 3:** List of ER stress-related genes with significant expression in SMMC-7721 cells (fold change ≥1.5-fold).

Probe set ID	Gene symbol	Control	G418	ATSE	1/2(G418 + ATSE)	ATSE/control
7964460	DDIT3	106	274	525	546	4.94
8076481	CYB5R3	262	209	539	279	2.06
8139003	HERPUD2	186	263	323	271	1.74
8042107	VRK2	536	973	884	1075	1.65
8156838	SEC61B	299	566	469	638	1.57
**8128650**	**SEC63**	**776**	**954**	**522**	**653**	−**1.50**
8062174	ERGIC3	984	1042	662	686	−1.50
7958644	ATP2A2	575	769	382	536	−1.50
7989619	PPIB	1595	2090	989	2036	−1.61
**8084634**	**DNAJB11**	**323**	**442**	**196**	**535**	−**1.62**
**7981335**	**HSP90AA1**	**1428**	**2906**	**831**	**2147**	−**1.72**
8178498	HLA-B	327	268	190	246	−1.72
7958130	HSP90B1	1426	2138	744	2022	−1.92
8091954	GOLIM4	641	378	329	364	−1.95
7983274	PDIA3	855	1271	437	949	−1.96
8023561	LMAN1	735	816	372	690	−1.98
7904881	PDIA3P	889	1360	440	980	−2.02
**8164165**	**GRP78**	**1317**	**2308**	**580**	**2346**	−**2.27**
8160914	VCP	333	318	114	278	−2.91

G418 (0.45 mg/mL); ATSE: *Akebia trifoliate* seed extract (0.625 mg/mL); 1/2(G418 + ATSE): a combination of half dose of each accordingly.

**Table 4:** List of genes with significant expression in the KEGG integrin-mediated cell adhesion pathway for HepG2 cells (fold change ≥1.5-fold).

Probe set ID	Gene symbol	Control	G418	ATSE	1/2(G418 + ATSE)	ATSE/control
7983763	MAPK6	233	314	382	405	1.64
8046380	ITGA6	216	323	385	220	1.78
8051670	SOS1	291	386	520	515	1.79
8084963	PAK2	173	252	360	338	2.08
8090162	ITGB5	330	357	220	150	−1.50
8111915	SEPP1	3337	2847	1761	1131	−1.87

G418 (0.45 mg/mL); ATSE: *Akebia trifoliate* seed extract (0.625 mg/mL); 1/2(G418 + ATSE): a combination of half dose of each accordingly.

**Table 5:** List of genes with significant expression in the KEGG-apoptosis pathway for HuH7 cells (fold change ≥1.5-fold).

Probe set ID	Gene symbol	Control	G418	ATSE	1/2(G418 + ATSE)	ATSE/control
8149733	TNFRSF10B	408	688	794	805	1.95
7966746	HRK	153	188	252	403	1.65
8065569	BCL2L1	1172	1558	1822	1293	1.55
8012257	TP53	212	209	320	66	1.51
7956989	MDM2	766	1242	1138	680	1.50
7924733	PARP1	102	90	67	56	−1.50

G418 (0.45 mg/mL); ATSE: *Akebia trifoliate* seed extract (0.625 mg/mL); 1/2(G418 + ATSE): a combination of half dose of each accordingly.

## Abbreviations

ATSE: * Akebia trifoliate* seed extract
HCC: Human hepatocellular carcinoma
TCM: Traditional Chinese medicine
MTT: 3-(4, 5-Dimethylthiazol-2-yl)-2, 5-diphenyltetrazolium bromide
ER: Endoplasmic reticulum
KEGG: Kyoto Encyclopedia of Genes and Genomes.

## References

[1] Venook A. P., Papandreou C., Furuse J., de Guevara L. L. (2010). The incidence and epidemiology of hepatocellular carcinoma: a global and regional perspective. *The Oncologist*.

[2] World Health Organization http://globocan.iarc.fr/Pages/fact_sheets_cancer.aspx.

[3] Mondal S., Bandyopadhyay S., Ghosh M. K., Mukhopadhyay S., Roy S., Mandal C. (2012). Natural products: Promising resources for cancer drug discovery. *Anti-Cancer Agents in Medicinal Chemistry*.

[4] Li Y., Martin R. C. G. (2011). Herbal medicine and hepatocellular carcinoma: applications and challenges. *Evidence-based Complementary and Alternative Medicine*.

[5] Wu P., Dugoua J. J., Eyawo O., Mills E. J. (2009). Traditional Chinese medicines in the treatment of hepatocellular cancers: a systematic review and meta-analysis. *Journal of Experimental and Clinical Cancer Research*.

[6] Wu M. C. (2003). Traditional Chinese medicine in prevention and treatment of liver cancer: function, status and existed problems. *Journal of Chinese Integrative Medicine*.

[7] Qi F. H., Li A. Y., Inagaki Y., Gao J., Li J., Kokudo N., Li X.-K., Tang W. (2010). Chinese herbal medicines as adjuvant treatment during chemoor radio-therapy for cancer. *BioScience Trends*.

[8] Li L., Yao X. H., Zhong C. H., Chen X. Z., Huang H. (2010). Akebia: a potential new fruit crop in China. *HortScience*.

[9] Chinese Pharmacopoeia Commission (2010). *China Pharmacopeia*.

[10] Qian B. W. (1987). *Application of Anti-Cancer Chinese Medicine*.

[11] Kang H. S., Kang J. S., Jeong W. S. (2010). Cytotoxic and apoptotic effects of saponins from akebia quinata on HepG2 Hepatocarcinoma cells. *Korean Journal of Food Preservation*.

[12] Jung H. J., Lee C. O., Lee K. T., Choi J., Park H. J. (2004). Structure-activity relationship of oleanane disaccharides isolated from Akebia quinata versus cytotoxicity against cancer cells and NO inhibition. *Biological and Pharmaceutical Bulletin*.

[13] Sun H., Fang W.-S., Wang W.-Z., Hu C. (2006). Structure-activity relationships of oleanane- and ursane-type triterpenoids. *Botanical Studies*.

[14] Fang Z. Q., Ren H. Y., Liang C. One kind of predictable Akebia Fructus extract in the treatment of primary liver cancer.

[15] Fang Z. Q., Ren H. Y., Liang C. One kind of predictable Akebia Fructus seed extract in the treatment of primary liver cancer.

[16] Fang Z. Q., Ren H. Y., Liang C. Akebia Fructus seed extractive and cytotoxin antibiotic combined composition and application.

[17] Fang Z. Q., Ren H. Y., Liang C. The mechanism of Akebia trifoliate seed inhibits the proliferation of liver cancer cell lines.

[18] Fang Z. Q., Ren H. Y., Liang C. (2013). The mechanism research on the proliferation inhibition by the common used herbal compound. *Chinese Journal of Basic Medicine in Traditional Chinese Medicine*.

[19] Li Y. J., Fang Z. Q., Tang C. L. (2003). Clinical epidemiological investigation and research of syndrome
distribution law in chinese medicine of 2060 cases of primary
liver carcinoma. *Chinese Medical Journal*.

[20] Chen D. S., Fang Z. Q. (2002). Analysis on the frequency of herbs used in primary hepatocellular carcinoma. *Liaoning Journal of Traditional Chinese Medicine*.

[21] Zhang W., Luo J. G., Zhang C., Kong L. Y. (2013). Different apoptotic effects of triterpenoid saponin-rich Gypsophila oldhamiana root extract on human hepatoma SMMC-7721 and normal human hepatic L02 cells. *Biological and Pharmaceutical Bulletin*.

[22] Podolak I., Galanty A., Sobolewska D. (2010). Saponins as cytotoxic agents: a review. *Phytochemistry Reviews*.

[23] Nieto-Miguel T., Fonteriz R. I., Vay L., Gajate C., López-Hernández S., Mollinedo F. (2007). Endoplasmic reticulum stress in the proapoptotic action of edelfosine in solid tumor cells. *Cancer Research*.

[24] Fribley A., Zeng Q., Wang C.-Y. (2004). Proteasome inhibitor PS-341 induces apoptosis through induction of endoplasmic reticulum stress-reactive oxygen species in head and neck squamous cell carcinoma cells. *Molecular and Cellular Biology*.

[25] Carracedo A., Gironella M., Lorente M., Garcia S., Guzmán M., Velasco G., Iovanna J. L. (2006). Cannabinoids induce apoptosis of pancreatic tumor cells via endoplasmic reticulum stress-related genes. *Cancer Research*.

[26] Berridge M. J. (2002). The endoplasmic reticulum: a multifunctional signaling organelle. *Cell Calcium*.

[27] Ron D., Walter P. (2007). Signal integration in the endoplasmic reticulum unfolded protein response. *Nature Reviews Molecular Cell Biology*.

[28] Chiu S. C., Huang S. Y., Chen S. P., Su C. C., Chiu T. L., Pang C. Y. (2013). Tanshinone IIA inhibits human prostate cancer cells growth by induction of endoplasmic reticulum stress in vitro and in vivo. *Prostate Cancer and Prostatic Diseases*.

[29] Xu Y., Chiu J. F., He Q. Y., Chen F. (2009). Tubeimoside-1 exerts cytotoxicity in heLa cells through mitochondrial dysfunction and endoplasmic reticulum stress pathways. *Journal of Proteome Research*.

[30] Velasco G., Verfaillie T., Salazar M., Agostinis P. (2010). Linking ER stress to autophagy: potential implications for cancer therapy. *International Journal of Cell Biology*.

[31] Ryuichi H., Miyahara K., Kawasaki T. (1972). Seed saponins of akebia quinata dence. I. hederagenin 3-0-glycosides. *Chemical and Pharmaceutical Bulletin*.

[32] Ryuichi H., Miyahara K., Kawasaki T. (1972). Seed saponins of Akebia quinata Dence. II. Hederagenin 3,28-0-bisglycosides. *Chemical and Pharmaceutical Bulletin*.

[33] Li Z. F., Wang Q., Liu Y. T. (2013). Chemical constituents from Akebia trifoliate. *Chinese Journal of Traditional Medical Formulae*.

[34] Jiang D., Shi S. P., Cao J. J., Gao Q. P., Tu P. F. (2008). Triterpene saponins from the fruits of Akebia quinata. *Biochemical Systematics and Ecology*.

